# Upcycling of Waste PVC into CaCO_3_/KOH-Modified Porous Carbon for Supercapacitor Applications

**DOI:** 10.3390/molecules30163420

**Published:** 2025-08-19

**Authors:** Wenbo Cai, Le Liu, Peng Zhang, Zhidan Lin

**Affiliations:** 1 Institute of Advanced Wear & Corrosion Resistant and Functional Materials, Jinan University, Guangzhou 510632, China; c15515073883@163.com (W.C.); 15037918177@163.com (L.L.); tzhangpeng@jnu.edu.cn (P.Z.); 2 National Joint Engineering Research Center of High Performance Metal Wear Resistant Materials Technology, Jinan University, Guangzhou 510632, China

**Keywords:** supercapacitors, chemical recycling, porous carbon materials, PVC plastic

## Abstract

This study introduces a green method for converting waste polyvinyl chloride (PVC) into hierarchical porous carbon materials. By using CaCO_3_ pre-activation to capture HCl and form meso/macroporous frameworks, followed by KOH activation to tune microporosity, high-surface-area porous carbon was successfully produced. The effects of KOH loading ratios (C-PVC:KOH = 1:1 to 1:3) on the primary activated carbon material were systematically investigated. It was found that a ratio of 1:2 (C-KOH-2) yielded optimal material properties, with a specific surface area of 1729 m^2^ g^−1^ and an oxygen doping content of 7.37%. Electrochemical measurements revealed that C-KOH-2 exhibited a high specific capacitance of 360.4 F g^−1^ at 1 A g^−1^, retaining 72.1% of its capacitance at 10 A g^−1^. The symmetric supercapacitors achieved an energy density of 9.9 Wh kg^−1^ at 125 W kg^−1^, with 93.12% capacitance retention over 5000 cycles. This dual-purpose approach enables the upcycling of PVC waste while promoting the development of high-performance electrodes.

## 1. Introduction

As living standards improve and the national economy grows, the consumption of non-renewable energy increases continuously, making it imperative to address the global energy crisis. Currently, the demand for energy storage devices is growing substantially in various fields like portable electronics, hybrid vehicles, and secondary energy systems [1,2]. Against this backdrop, electric double-layer capacitors (EDLCs) [3,4] have emerged as a prominent type of supercapacitors (SCs) [5,6], considering their excellent performance in charge–discharge kinetics, power density, and cycling stability.

At present, the use of porous carbon materials [7,8,9,10,11] is regarded as a leading solution to advanced electrode applications due to their cost-effectiveness, exceptionally high electrochemical stability, tunable porous structures, and superior conductivity. However, their large-scale commercialization is severely impeded by their relatively low energy density and specific capacitance. An effective solution to this problem is to develop advanced porous carbons with precisely controllable morphology and pore architectures. Among them [12], hierarchical porous architectures are promising to improve the capacitance and rate capability of EDLCs, which is attributed to a rational arrangement of micropores (<2 nm), mesopores (2–50 nm), and macropores (>50 nm) [13]. By providing a high specific surface area for ion adsorption, micropores enhance charge storage capacity. Also, they promote efficient ion diffusion and transport, acting as “ion buffer reservoirs” to shorten migration pathways. Notably, a strategy has been demonstrated to optimize the performance of porous carbon-based supercapacitors, that is, the heteroatom doping (O [14], N [15], S [16], B [17], and P [18]) into the carbon framework. Due to their smaller atomic radius, oxygen atoms can be incorporated easily into the carbon structure. Due to their high electronegativity, the formed C-O bonds significantly enhance the surface polarity of the material [19]. Also, oxygen-containing functional groups (such as quinone moieties) could participate in redox reactions. Apart from enabling the improvement of surface wettability via oxygen doping, this also enhances pseudocapacitance through reversible redox processes. Due to their unique multi-level pore channels and optimized pore size distribution, hierarchical porous materials can produce an outstanding electrochemical performance in EDLC applications. However, their large-scale practical application is severely hindered by complex synthesis and high production costs.

As one of the most common polymers found in daily use, polyvinyl chloride (PVC) is widely applied in the packaging and construction sectors for manufacturing pipes, roofing materials, flooring, and cable insulators [20,21,22]. However, PVC waste tends to accumulate in the environment over time if not effectively recycled and managed, which causes not only severe pollution but also the degradation of soil, water bodies, and ecosystems. The conversion of plastic waste into high-value carbon materials resolves the environmental issues induced by discarded plastics, representing an effective solution to producing porous carbon materials [23,24,25]. However, the equipment could be corroded as the high chlorine content in PVC plastic reduces thermal stability and causes the release of HCl. For the improved stability of PVC plastic during production, it is usually necessary to introduce excessive amounts of plasticizers (such as phthalates) and thermal stabilizers (such as lead salts and calcium-zinc composites). Exhibiting significant chemical inertness in subsequent recycling processes, these additives render solvothermal [26,27,28] or mechanical grinding [29] methods ineffective for the efficient decomposition of the PVC matrix. Consequently, the recycling efficiency is low. Moreover, environmental pollution is exacerbated by the use of toxic organic solvents such as polyethylene glycol and DMF. Therefore, it is crucial to develop an efficient approach to plastic recycling and utilization.

In this study, a CaCO_3_/KOH synergistic activation strategy is applied [30,31], involving cost-efficient CaCO_3_. At high temperatures, CaCO_3_ decomposes into CaO and CO_2_, with calcium components used to capture HCl from PVC pyrolysis for reduced corrosion. Meanwhile, evolved CO_2_ etches mesopores via gas-phase activation, with nano-CaO particles as sacrificial hard templates [32]. Subsequently, KOH activation gives rise to micropores, with hierarchical porous networks established as a result [33,34,35]. During this process, carbon surfaces are concurrently oxidized, with oxygen-containing functional groups introduced for enhanced electrochemical performance [36,37]. This study introduces a new method of converting waste PVC into carbon materials for sustainable supercapacitors.

## 2. Results and Discussion

### 2.1. Influence of Different Activation Methods on the Microstructure of Porous Carbon Materials

**Figure 1:**
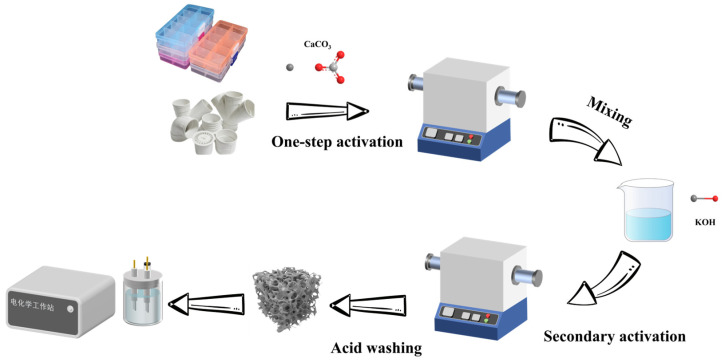
Synthesis of porous carbon materials.

The porous carbon synthesis procedure and subsequent analysis of porous carbon materials are illustrated in Figure 1.

**Figure 2:**
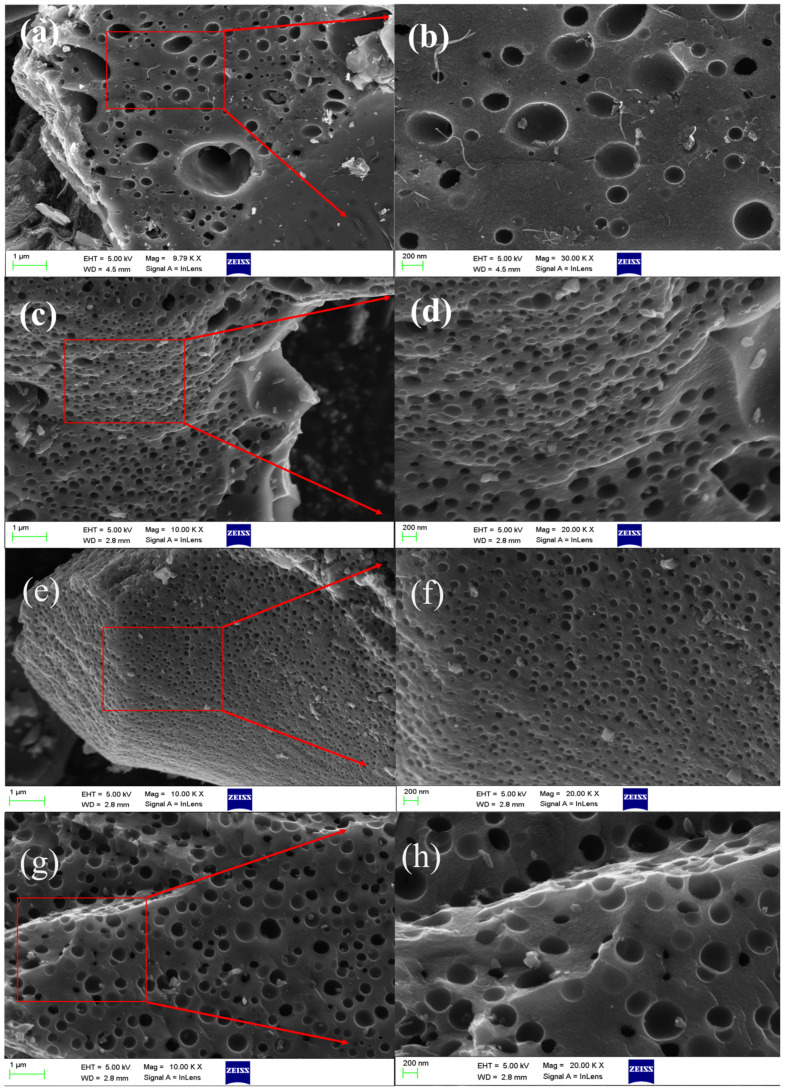
SEM images of C-KOH-0 (**a**,**b**), C-KOH-1 (**c**,**d**), C-KOH-2 (**e**,**f**), and C-KOH-3 (**g**,**h**).

Figure 2a–h displays Scanning Electron Microscopy (SEM) images of carbon materials that were prepared with varying KOH ratios. Carbon materials from the primary pyrolysis of PVC and calcium carbonate are illustrated in SEM micrographs in Figure 2a,b. The SEM analysis reveals sparse, irregular macropores (pore diameters ranging from 50 to 200 nm) with a low lacuna density on the surface. The pore-forming mechanism of calcium carbonate is characterized by the high-temperature carbonization decomposition of the material, which results in the release of CO_2_ gas and the formation of initial pores (Equation (1)). In the interim, the calcium-based by-products dissolved during acid washing, which further shapes an interconnected mesoporous framework (Equations (2) and (3)).

C + CO_2_ → 2CO (1)

CaCO_3_ + 2HCl → CaCl_2_ + CO_2_ + H_2_O (2)

CaO + 2HCl → CaCl_2_ + H_2_O (3)

After secondary activation with KOH, the carbon materials exhibited a honeycomb-like morphology, as shown in Figure 2c–h. Unlike the C-KOH-0 sample, these materials feature more uniform and dense pore networks, with a homogeneous distribution of pore sizes. From the samples C-KOH-1 (Figure 2c,d), the material’s surface significantly promotes pore development following secondary activation in comparison to single-step activation. This enhances charge storage capacity and reaction kinetics by increasing the specific surface area of the carbon material by exposing more electrochemically active sites, such as edge defects and heteroatom functional groups, to the electrode/electrolyte interface through optimization of the pore structure. C-KOH-2 (Figure 2e,f) exhibited a porous carbon structure with denser, more uniform, and ordered pore arrays. As the KOH loading rises, Figure 2g,h shows that the pore density on the surface of C-KOH-3 does not increase further; instead, it declines. The sharp, angular cuts of the carbon edges are indicative of the aggressive etching of the framework by the excess KOH, which caused the existing micropores to coalesce or collapse into mesopores and macropores.

6KOH + 2C → 2K_2_CO_3_ + 3H_2_ + 2K (4)

K_2_CO_3_ → K_2_O + CO_2_ (5)

This may impair the continuity of the carbon skeleton due to over-activation, which impedes ion diffusion and reduces the specific surface area. As illustrated in the figure, KOH functions as an activator and exhibits a threshold during the secondary activation process. The ion transport and the exposure of active sites are significantly influenced by the appropriate proportion of the layered pore structure. Among all samples, C-KOH-2 exhibits the most homogeneous distribution and the richest pore structure.

**Figure 3:**
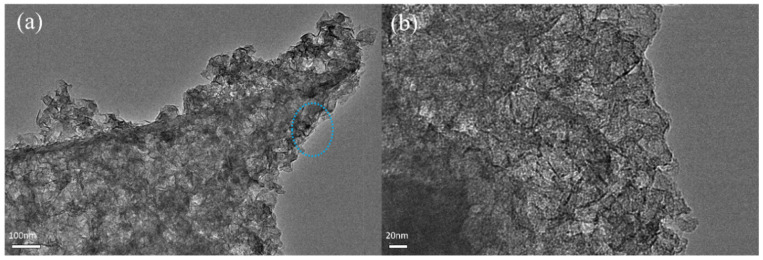
TEM images (**a**,**b**) of sample C-KOH-X (X = 2).

The microstructure of C-KOH-2 was characterized by high-resolution transmission electron microscopy (HRTEM). Figure 3b is an enlarged view of the selected area in Figure 3a, which further substantiates the highly open pore network characteristics. This pore network optimizes ion diffusion paths and electron conduction networks, thereby offering significant benefits for improving the kinetics of electrochemical energy storage [38].

### 2.2. Effect of the Two Activation Methods on the Textural Properties of Porous Carbon Materials

**Figure 4:**
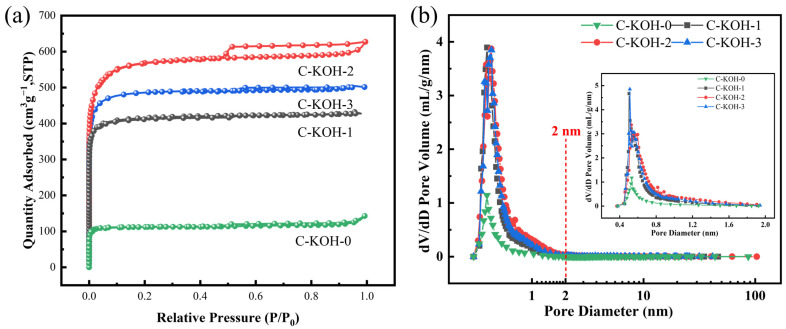
Adsorption and desorption curves and pore size distribution for all samples (**a**,**b**).

The specific surface area and pore size distribution of the samples were determined by analyzing their N_2_ adsorption–desorption isotherms using the Brunauer-Emmett-Teller (BET) method, with complementary analyses via multi-point BET (specific surface area calculation), Barrett-Joyner-Halenda (BJH) method (mesopore size distribution), and Horváth-Kawazoe (H-K) method for micropore analysis (inset). As illustrated in Figure 4a,b, all samples in the C-KOH-X (X = 0, 1, 2, 3) series exhibit a hybrid isotherm characteristic, combining features of Type I and Type IV. A rapid increase in adsorption capacity at low relative pressures (*P*/*P*_0_ < 0.1) indicates the presence of microporous structures in the carbon materials. Subsequently, a further rise in adsorption capacity at high relative pressures (*P*/*P*_0_ > 0.8) suggests the existence of mesoporous characteristics, which is consistent with the results of average pore size analysis. As illustrated in Table 1, sample C-KOH-0 exhibits an average pore diameter of 3.67 nm, substantially exceeding that of the secondary-activated carbon. A micropore volume of 0.12 cm^3^ g^−1^ and an additional pore volume of 0.09 cm^3^ g^−1^ were identified, which indicates that mesopores and macropores are the predominant pore types in the calcium–carbonate-activated material. In addition, the micropore fraction is significantly increased by the secondary activation.

The specific surface areas of C-KOH-1, C-KOH-2, C-KOH-3, and C-KOH-0 are 1269, 1729, 1453, and 326 m^2^ g^−1^, respectively, as illustrated in Table 1. The specific surface area of carbon materials is significantly increased by the introduction of KOH for secondary activation. The micropore volume increases considerably from 0.12 to 0.88 cm^3^ g^−1^ from C-KOH-0 to C-KOH-2, indicating that the micropores formed by KOH etching are the primary factor contributing to the increase in specific surface area. Nevertheless, the specific surface area decreases in comparison to that of C-KOH-2 in the case of over-activation (C-KOH-3), despite the micropore volume remaining at 0.71 cm^3^ g^−1^. This reduction in effective surface area may be attributed to micropore coalescence or structural disordering.

In summary, sample C-KOH-2 has a high specific surface area and suitable micro- and mesoporous structures. A high specific surface area means there are more active sites on the surface. This increases the charge storage capacity of the electrode material and promotes interfacial reactions. Additionally, the optimized layered pore structure improves mass transfer, enhancing the material’s rate performance [39].

### 2.3. Effect of Activation Method on the Chemical Composition of Porous Carbon Materials

**Figure 5:**
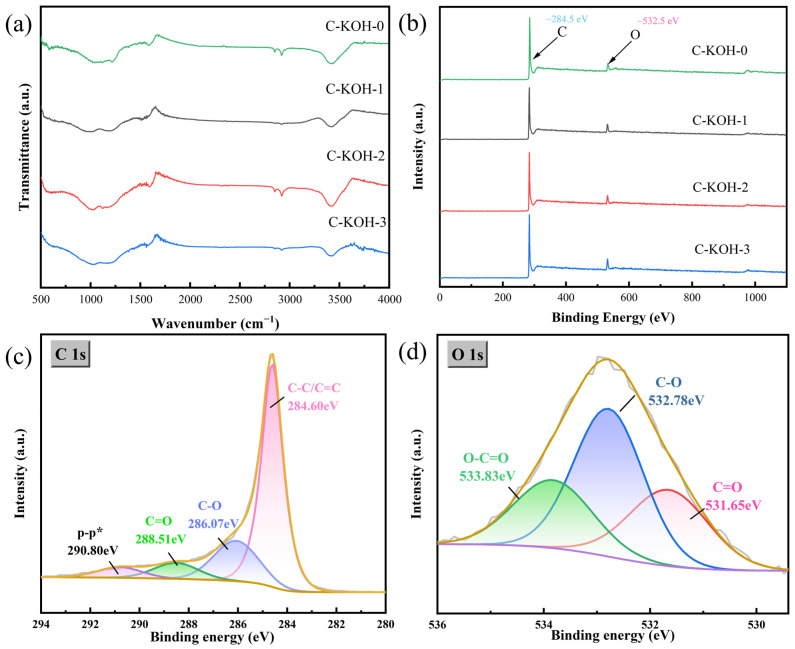
(**a**) IR spectra of samples, (**b**) full spectrum XPS of samples, (**c**) fine spectrum of C 1s for C-KOH-2 material, and (**d**) fine spectrum of O 1s for C-KOH-2 material.

The functional groups in the materials were characterized using Fourier transform infrared (FTIR) spectroscopy. The O-H stretching vibrations are represented by a broad absorption peak that is observed between 3400 and 3500 cm^−1^, as illustrated in Figure 5a. Furthermore, the absorption peaks in the 1000–1300 cm^−1^ region are correlated with C=O groups (originating from hydroxyl/ether/ester moieties), C-C, and C-O [40]. The findings indicate that the activated samples possess functional groups that contain oxygen in comparable quantities. This modification is expected to improve the surface wettability of the materials, which in turn optimises their electrochemical behaviour and facilitates interfacial reaction kinetics in aqueous electrolytes.

Figure 5b shows distinct C 1s (~284.5 eV) and O 1s (~532.5 eV) peaks in C-KOH-X (X = 0, 1, 2, 3), confirming oxygen doping. Meanwhile, no chlorine element was detected. Quantitative analysis indicates that primary carbonization with only a CaCO_3_ template yielded a low oxygen content of 6.51%, mainly from residual oxygen-containing groups from PVC carbonization and surface-adsorbed oxygen during CaCO_3_ decomposition. In contrast, secondary KOH activation greatly increases oxygen content, which rises with the KOH ratio, reaching 5.35%, 7.37%, and 8.27% for C-KOH-X (X = 1, 2, 3), respectively, as listed in Table 2.

Peak fitting analysis of the X-ray photoelectron spectroscopy (XPS) spectrum of sample C-KOH-2 was used to investigate the chemical states of the C and O elements in the sample. The analysis yielded distinct C-C/C=C bond peaks (284.60 eV), C-O bond peaks (286.07 eV), and C=O bond peaks (288.51 eV) in the C 1s spectrum (Figure 5c). Similarly, analysis of the O 1s spectrum (Figure 5d) identified three components: C=O (~531.65 eV), C-O (~532.78 eV), and O=C-O (~533.83 eV) [41]. The introduction of oxygen can affect the electrochemical properties of materials. Hydroxyl (-OH) and carboxyl (-COOH) groups enhance the hydrophilicity of materials, facilitating electrolyte penetration and shortening the ion diffusion path. Moreover, quinone (C=O) and carboxyl groups enable reversible redox reactions during charging and discharging. These reactions provide additional pseudocapacitance and activate defect sites, improving charge storage.

C=O + H^+^ + e^−^ ↔ C-OH (6)

O-C=O + H^+^ + e^−^ ↔ HO-C-O (7)

In summary, the CaCO_3_/KOH activation process optimizes material wettability, pseudocapacitive activity, and electron transport efficiency through oxygen incorporation.

**Figure 6:**
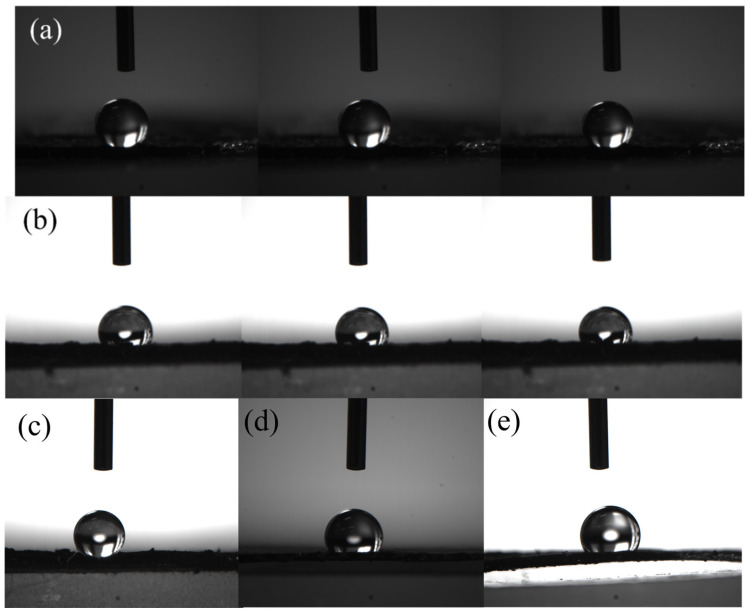
Contact angle comparison between commercial carbon and C-KOH-2 (**a**,**b**), and for C-KOH-0, C-KOH-1, and C-KOH-3 (**c**–**e**).

To illustrate the effect of oxygen doping, contact angle measurements were conducted under identical experimental conditions on a commercial activated carbon sample (with a carbon content of >98%) and the as-prepared C-KOH-2 sample. Figure 6a shows that the commercial carbon material has a significantly larger contact angle. Figure 6b highlights a striking disparity in contact angle measurements. The oxygen-doped C-KOH-2 has an average contact angle of 115.4°, much lower than the commercial counterpart’s 145.1°. Furthermore, the contact angles measured across the sample series were consistently lower than those of commercial carbon materials, with values as follows: 132.4° for C-KOH-0, 125.1° for C-KOH-1, and 115.0° for C-KOH-3 (Figure 6c–e). The contact angle exhibited a distinct monotonic decrease as the oxygen content increased.

Oxygen doping introduces hydrophilic groups. This significantly enhances surface polarity and electrolyte wettability, as demonstrated by the reduction of the contact angle by ~35°. This hydrophilicity optimization improves the electrode/electrolyte interfacial reaction kinetics [42]. It enables more thorough electrolyte penetration in the oxygen-doped material and reduces charge transfer resistance.

### 2.4. Characterization of Graphitization Degree and Crystalline Structure of Porous Carbon Materials

**Figure 7:**
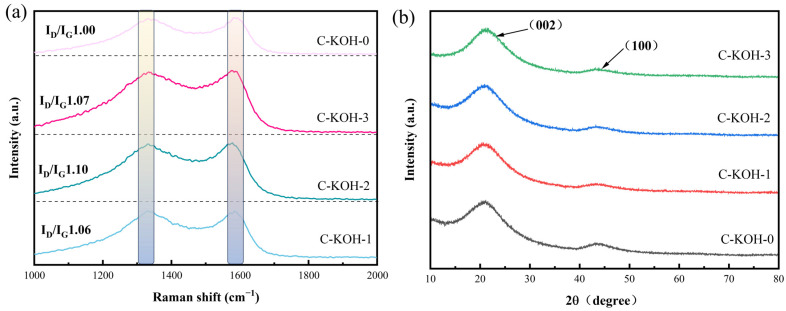
(**a**) Raman plots and (**b**) XRD plots of C-KOH-X (X = 0, 1, 2, 3).

The structural features of the synthesized porous carbon with varying KOH ratios are revealed by the Raman spectra and X-ray diffractograms (Figure 7a,b). The Raman spectrum displays a D band at ~1350 cm^−1^, associated with sp^3^-hybridized carbon atoms or structural defects such as pore edges, vacancies, and heteroatom doping. The intensity of this band reflects the degree of structural disorder or defect density. The G band at approximately 1580 cm^−1^ is associated with in-plane vibrations of sp^2^-hybridized graphite microcrystals, reflecting the orderliness of the carbon framework [14]. A higher G-band intensity suggests greater graphitization and more regular carbon layer stacking. Raman band fitting shows that the I_D_/I_G_ intensity ratios of C-KOH-X (X = 0, 1, 2, 3) were 1.00, 1.06, 1.10, and 1.07, respectively. Notably, C-KOH-0 has a higher degree of graphitization than the activated samples, while C-KOH-2 shows the highest I_D_/I_G_ ratio among the KOH-activated materials, indicating increased structural defects and disorder. Through the generation of active surface sites (dangling bonds and edge carbons), these defects expand the electrode–electrolyte contact area, leading to superior electrochemical behavior.

The XRD patterns in Figure 7b demonstrated a moderate-intensity diffraction peak at 2θ = 20–25°, which was related to the (002) crystal plane of graphitic carbon and a weakened peak at 2θ = 40–45°, which was related to the (100) plane. These broad peaks indicate that all samples are predominantly amorphous carbon with disordered structures. Compared to C-KOH-0, the (002) peaks of the KOH-activated samples (C-KOH-X, X = 1, 2, 3) are broader and less intense, suggesting reduced graphitization and structural order. This disorder is due to the pores and defect sites introduced by KOH activation. The degree of graphitization affects the electrical conductivity and structural regularity of carbon materials, highlighting a trade-off between porosity and graphitic order during activation [43].

## 3. Electrochemical Performance Evaluation of Carbon Materials Prepared by Different Activation Methods

### 3.1. Electrochemical Properties of CaCO_3_ Activation

**Figure 8:**
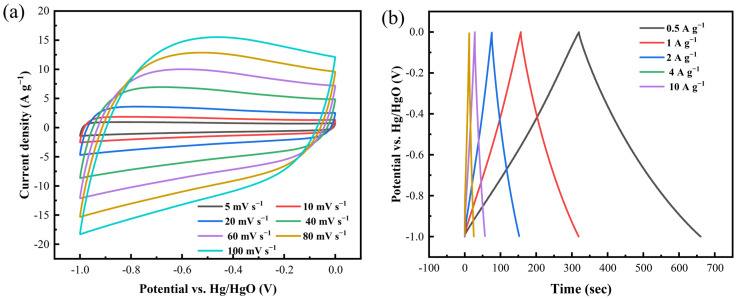
CV, GCD plots for calcium carbonate activated carbon materials (**a**,**b**).

Cyclic voltammetry (CV) and galvanostatic charge–discharge (GCD) measurements were employed to characterize the electrochemical performance of carbon materials that had been treated with CaCO_3_. As shown in Figure 8a, the carbon material’s CV curve is roughly rectangular at low scan rates, indicating some reversible capacitance. Figure 8b displays GCD curves with an isosceles triangular shape, typical of a double-layer capacitor [44]. The material’s specific capacitance was calculated to be 160.7 F g^−1^ at 1 A g^−1^, suggesting room for improvement.

### 3.2. Synergistic-Activated Carbon Electrochemical Testing

**Figure 9:**
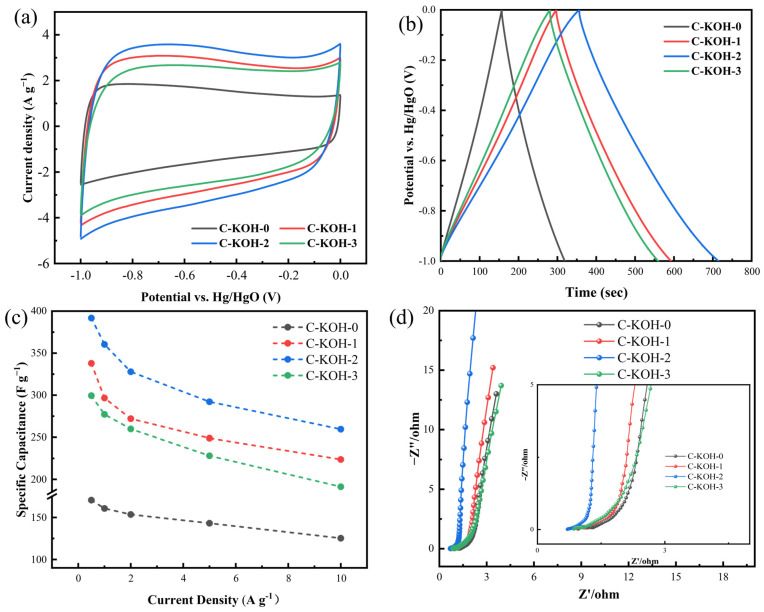
For samples C-KOH-X (X = 0, 1, 2, 3): (**a**) CV curves at a scan rate of 10 mV s^−1^, (**b**) GCD curves at a current density of 1 A g^−1^, (**c**) specific capacities, and (**d**) Nyquist plots of C-KOH-X.

Figure 9a shows the CV curves of different materials at a scan rate of 10 mV s^−1^. All carbon electrodes display nearly rectangular CV profiles, indicating good electric double-layer capacitive behavior. Among them, C-KOH-0 has the smallest CV curve integral area, while C-KOH-2 has the largest, showing the highest charge storage capacity at this scan rate. Compared to single-activated C-KOH-0, secondary KOH activation greatly boosted energy storage performance: The CV curve area increased significantly as the KOH ratio rose from 1:1 to 1:2 but decreased when further increased to 1:3. This aligns with the SEM and BET results, confirming that the specific surface area improvement boosts specific capacitance by providing more active sites for ion adsorption. Figure 9b presents the GCD curves at 1 A g^−1^, showing isosceles triangular shapes that reflect excellent charge-discharge reversibility.

The specific capacitances of samples C-KOH-X (X = 0, 1, 2, 3) were calculated to be 160.7, 296.6, 360.4, and 277.2 F g^−1^. The specific capacitances of C-KOH-X (X = 1, 2, 3) all exceed those of the commercial YEC-8A (260.1 F g^−1^). These values are in good accordance with the energy storage performance deduced from the CV curves. Notably, C-KOH-2 exhibits the highest charge storage capability among all samples. Figure 9c depicts the specific capacitances of the samples at different current densities. C-KOH-2 showed the best performance. Even under a high current density of 10 A g^−1^, the material maintains a specific capacitance of 259.5 F g^−1^, equivalent to 72.1% capacitance retention. Figure 9d displays the Nyquist plot of C-KOH-2. In the low-frequency domain, the plot exhibits a steeper trend and is nearly parallel to the imaginary axis, demonstrating exceptional charge transport characteristics [45,46].

**Figure 10:**
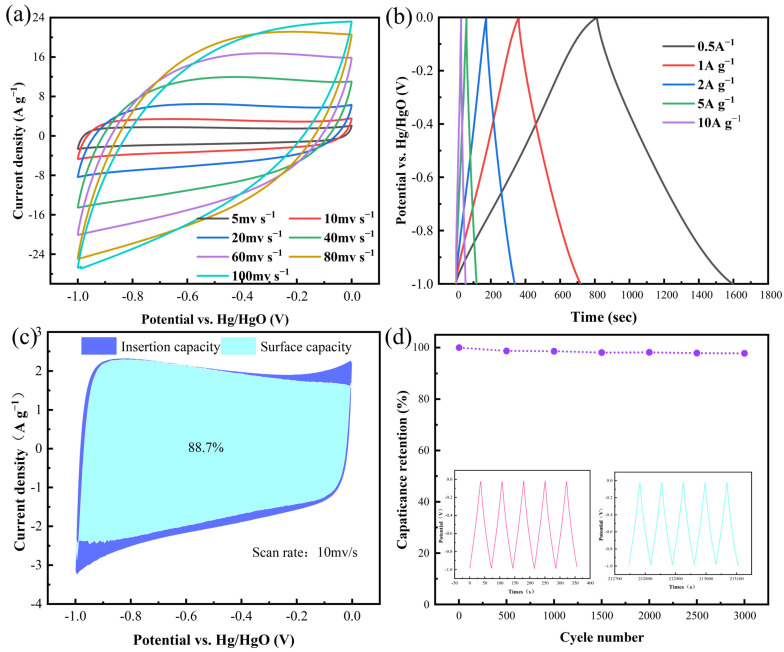
(**a**) CV plots of C-KOH-2 with different scan rates, (**b**) GCD with different current densities, (**c**) surface effect capacitance and diffusion capacitance contributions, and (**d**) 3000 charge–discharge cycles.

Building upon these findings, a comprehensive evaluation of the electrochemical energy-storage performance of C-KOH-2 was conducted. CV tests (Figure 10a) show that between 10 and 100 mV s^−1^, its CV curves are roughly rectangular without distinct redox peaks, indicating the material mainly uses fast-reversible electric double-layer capacitance. GCD measurements show that between 0.5 and 10 A g^−1^, the GCD curves (Figure 10b) are highly symmetric isosceles triangles with an IR drop <0.05 V, showing very low equivalent series resistance and excellent rate performance. As shown in Figure 10c, at a scan rate as low as 10 mV s^−1^, capacity contribution analysis shows that surface adsorption-dominated capacity makes up 88.7%. Figure 10d shows that after 3000 charge–discharge cycles at 8 A g^−1^, the C-KOH-2 electrode retains 97.81% of its specific capacitance. This cycling durability is attributed to abundant mesopores and micropores that effectively alleviate volume strain from ion insertion/extraction, along with moderate surface functionalization (C-O, C=O groups) that enhances electrode–electrolyte interfacial compatibility.

### 3.3. Electrochemical Performance Analysis of Symmetric Supercapacitors

A symmetrical supercapacitor was constructed with a PAA/KOH gel electrolyte to confirm the practical applicability of the C-KOH-2 electrode.

**Figure 11:**
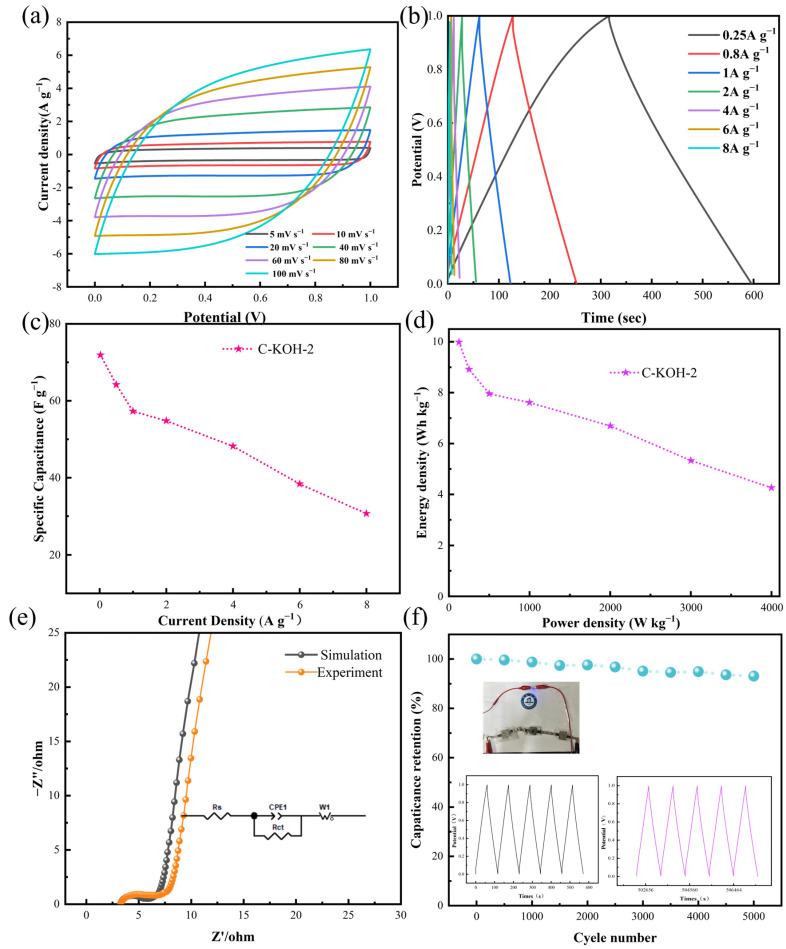
Symmetric supercapacitor C-KOH-2 (**a**) GCD curves, (**b**) CV curves, (**c**) specific capacitance values, (**d**) Ragone plots of samples, (**e**) Nyquist plots, and (**f**) capacitance retention rates of 5000 cycles of charge and discharge of samples with blue LED bulbs lit in series.

As depicted in Figure 11a, the curve exhibited a typical rectangular shape at a low scan rate of 5 mV s^−1^. Even at a high scan rate of 100 mV s^−1^, the curve maintains this characteristic, demonstrating the material’s rapid charge storage capability under high-frequency conditions. Galvanostatic charge–discharge profiles spanning 0.25–8 A g^−1^ (Figure 11b) confirm symmetric isosceles triangular shapes for the C-KOH-2 electrode. This material achieves 71.9 F g^−1^ at 0.25 A g^−1^ (Figure 11c). Ragone analysis (Figure 11d) indicates energy densities of 9.9 Wh kg^−1^ (125 W kg^−1^) and 7.6 Wh kg^−1^ at 1000 W kg^−1^. The EIS spectrum (Figure 11e) shows an excellent fit. The nearly vertical line in the low-frequency region indicates ideal capacitive behavior, and the high-frequency semicircle corresponds to a charge-transfer resistance of 0.87 Ω. Long-cycle stability tests (Figure 11f) demonstrate that after 5000 cycles at 1 A g^−1^, the device retains 93.12% of its initial capacity, with GCD curves showing high similarity before and after cycling, reflecting structural stability under double-layer storage mechanisms. The inset illustrated a practical application: Three series-connected C-KOH-2 supercapacitors powered a standard blue LED, providing experimental evidence for applications in flexible electronics and emergency power supplies.

## 4. Materials and Methods

### 4.1. Materials Used in This Experiment

PVC products derive from routine collection in daily life (an organizer with plastic tubes). Calcium carbonate and potassium hydroxide were procured from Shanghai Macklin Biochemical Technology Co., Ltd. (Shanghai, China). High-purity nitrogen gas was obtained from Guangzhou Damao Gas Company (Tianjin, China). Acetylene black was purchased from Denka Company Limited (Tokyo, Japan). Polytetrafluoroethylene (PTFE) emulsion was purchased from Daikin Industries, Ltd. (Osaka, Japan). The activated carbon material was purchased from Wenyan Technology Co., Ltd. (Hangzhou, China). YEC-8A activated carbon was purchased from Fuzhou Yi Huan Carbon Co., Ltd. (Fuzhou, China).

### 4.2. Material Preparation

#### 4.2.1. Preparation of Porous Carbon Materials Derived from PVC as the Carbon Source

During the preparation of porous carbon, the two types of PVC plastic were rinsed with deionized water first. Then, they were vacuum-dried at 60 °C for 12 h to obtain clean PVC fragments. The dried PVC was blended with CaCO_3_ at a mass ratio of 1:1.5 using a crusher to form a homogeneous powder mixture. The mixture was charged into a tube furnace and underwent N_2_ purging at a flow rate of 50 mL min^−1^, followed by heating to 800 °C at a rate of 10 °C min^−1^. After an hour-long isothermal hold, the system was allowed to cool naturally to room temperature. The resulting material was then purified using 1 M HCl to eliminate the CaCO_3_ impurities. After the acid treatment, the material was rinsed thoroughly with deionized water until a neutral pH was achieved. Finally, the product underwent vacuum drying at 80 °C for 12 h, yielding the primary carbon material, C-PVC.

To functionalize the pristine carbon, it was divided into three 1 g portions. Each portion was then combined with solid KOH at mass ratios of 1:1, 1:2, and 1:3. Each blend was mixed with 10 mL of deionized water while stirring to create homogeneous slurries. After oven-drying at 100 °C for 4 h, KOH-impregnated precursors were obtained. The precursors underwent pyrolysis in a tube furnace with a 50 mL/min N_2_ flow rate, using a 5 °C/min ramp to 800 °C and a 2-h isothermal treatment. After cooling in the furnace, the products were immersed in 1 M HCl, rinsed to a neutral pH, and dried at 80 °C for 12 h. The resulting samples were labeled C-KOH-1, C-KOH-2, and C-KOH-3. For comparison, the sample without KOH treatment was labeled C-KOH-0.

#### 4.2.2. Material Characterization

Surface morphology was observed by scanning electron microscopy (SEM, ULTRA 55, Oberkochen, Germany) at 5 kV accelerating voltage. Microstructure was examined using high-resolution transmission electron microscopy (HRTEM, JEOL JEM-2100F Tokyo, Japan) at 80 kV. Phase composition was characterized by X-ray diffraction (XRD, Ultima IV, Tokyo, Japan) with Cu-Kα radiation (λ = 1.5418 Å), 40 kV voltage, 40 mA current, scanning from 10° to 80° at 5° min^−1^. Chemical states and surface elements were analyzed by X-ray photoelectron spectroscopy (XPS, Escalab 250Xi, Waltham, MA, USA) using Al Kα radiation (0–1400 eV). Binding energies were referenced to the adventitious C 1s peak at 284.6 eV. Molecular structures were characterized using Fourier transform infrared spectroscopy (FTIR, Nicolet iS50, Waltham, MA, USA) with KBr pellets at 4 cm^−1^ resolution (32 scans, 500–4000 cm^−1^). Specific surface area and pore size distribution were determined with a 3H-2000PS2 (Beijing, China) analyzer after degassing at 200 °C for 1440 min. Structural ordering and defect density were assessed by Raman spectroscopy (Raman, LabRAM HR Evolution, Paris, France) using 532 nm excitation (1000–2000 cm^−1^). Contact angles were measured via the sessile drop method (SDC-100s, SINDIN, Dongguan, China) with 5 μL ultrapure water (10 s equilibration). Electrochemical impedance spectroscopy (CH Instruments, Shanghai, China) was performed on a CHI 760E system (5 mV amplitude, 0.01 Hz–100 kHz frequency range).

#### 4.2.3. Electrochemical Performance Test

The accurate determination of the specific gravimetric capacitance of supercapacitors is made possible by the processing of the galvanostatic charge–discharge (GCD) profiles.

(8) $$C_{m}=\frac{I∆t}{m∆V}$$

In this expression, *C_m_* (F g^−1^) denotes the specific capacitance of the single-electrode material, *I* (A) the applied discharge current, *m* (g) the mass of the active material loaded on the working electrode, Δ*t* (s) the discharge duration extracted from the galvanostatic profile, and Δ*V* (V) the operating potential window of the individual electrode.

Energy and power densities are the key practical metrics for electrode materials. The gravimetric energy density (Wh kg^−1^) is obtained from *E* = $\frac{1}{2}$ *C*(Δ*V*)^2^ using the parameters defined in Equation (9).


(9) $$E=\frac{I\times ∆V\times ∆t}{2\times m\times 3.6}$$

*I* (A): discharge current; Δ*t* (s): discharge time; and Δ*V* (V): linear voltage window (*V_initial_*–*V_final_*); *m* (g): active mass.

The gravimetric power density (W kg^−1^) denotes the maximum deliverable power per unit mass.

(10) $$P=\frac{3600E}{∆t}$$

where *E* is the energy density, and Δ*t* denotes the discharge duration.

## 5. Conclusions

This study synthesized hierarchical porous carbon materials with microporous–mesoporous structures through a two-step activation process and applied them to supercapacitors. The initial activation process utilized calcium carbonate as an activator, absorbing and immobilizing elemental chlorine and reducing contamination of the equipment materials. Concurrently, mesoporous carbon materials were prepared. Subsequently, potassium hydroxide was incorporated during the second activation process to encourage the development of microporous structures. Sample C-KOH-2 had a maximum specific surface area of 1729 m^2^ g^−1^. Compared to single CaCO_3_ activation, the combined activation approach significantly enhanced the electrochemical performance of the carbon material, and the performance varied with different KOH ratios. C-KOH-2 exhibited the best performance, with a specific capacitance of 360.4 F g^−1^ at 1 A g^−1^. Furthermore, a symmetric supercapacitor with a PAA/KOH gel electrolyte was fabricated. The supercapacitor based on C-KOH-2 demonstrated a capacitance of 71.9 F g^−1^ at a current density of 0.25 A g^−1^ and an energy density of 9.9 Wh kg^−1^ at a power density of 125 W kg^−1^. It also demonstrated outstanding cycling stability, maintaining 93.12% of its initial capacitance after 5000 charge–discharge cycles at 1 A g^−1^.

## Figures and Tables

**Table 1:** Specific surface area and pore size distribution data of C-KOH-X (X = 1, 2, 3, 0).

Samples	*S_BET_* (m^2^ g^−1^)	*V_total_* (cm^3^ g^−1^)	*V_Micro_* (cm^3^ g^−1^)	*V_Meso+Macro_* (cm^3^ g^−1^)	*D_ap_* (nm)
C-KOH-1	1269	0.66	0.59	0.07	2.35
C-KOH-2	1729	0.96	0.88	0.08	2.22
C-KOH-3	1453	0.78	0.71	0.07	2.14
C-KOH-0	326	0.21	0.12	0.09	3.67

*S_BET_*: specific surface area; *V_total_*: total pore volume; and *D_ap_*: average pore diameter.

**Table 2:** Elemental content diagrams for C-KOH-X (X = 1, 2, 3, 0) (%).

Element	C	O
C-KOH-1	92.41	6.35
C-KOH-2	92.11	7.37
C-KOH-3	90.89	8.27
C-KOH-0	94.10	5.51

## Data Availability

The data presented in this study are available upon request from the corresponding author.
